# 

**Published:** 1903-11-15

**Authors:** 


### ANNOUNCEMENTS.

THE thirty-eighth annual meeting of the Ohio State Dental Society will be held at the great Southern Hotel in Columbus, Ohio, December 1, 2 and 3, 1903. An excellent program is being prepared and an effort is to be made to get out the largest attendance the society has ever had. Make your arrangements to attend this meeting.

THE American Society of Orthodontists will hold its annual meeting in Buffalo, N. Y., this year, beginning December 31st and continuing for three days. A splendid program has been prepared, and every one interested in this subject should make his plans to attend this meeting.

---

Program of the meeting of the Institute of Dental Pedagogics, to be held in Buffalo, December 28th, 29th and 30th, at the Iroquois Hotel. All interested in education and the elevation of the standards of the dental colleges are very earnestly requested to attend this meeting.

1. President's Address. Some Faults of the Prevailing Dental Training, Dr. J. D. Patterson, Kansas City. Discussion to be opened by Dr. John I. Hart, New York; Dr. B. Holly Smith, Baltimore; Dr. H. P. Carlton, San Francisco; Dr. Geo. E. Hunt, Indianapolis.

2. Prosthesis. Two papers. (a) Methods of Teaching the Artistic Elements of Prosthetic Dentistry, Dr. A. O. Hunt, Omaha, Neb. (b) Methods of Teaching the Anatomical Arrangements of Teeth, Dr. B. J. Cigrand, Chicago. Discussion to be opened by Dr. N. S. Hoff, Ann Arbor; Dr. G. H. Wilson, Cleveland; Dr. R. R. Freeman, Nashville; Dr. F. H. Berry, Milwaukee.

3. An Ideal in Pathology. Paper by D. R. Stubblefield, Nashville. Discussion to be opened by Dr. H. A. Smith, Cincinnati; Dr. T. B. Hartzell, Minneapolis; Dr. A. H. Peck, Chicago; Dr. O. L. Hertig, Pittsburg.

4. Orthodontia Technology. Two papers. Dr. S. H. Guilford, Philadelphia, Dr. C. S. Case, Chicago. Discussion to be opened by Dr. W. E. Grant, Louisville; Dr. A. E. Webster, Toronto; Dr. H. B. Pullen, Buffalo; Dr. H. T. Smith, Cincinnati.

5. The Value of Instruction in Dental History and Literature. Paper by H, L. Ambler, Cleveland. Discussion to be opened by Dr. Charles McManus, Hartford; Dr. H. J. Kennerly, St. Louis; Dr. B. J. Cigrand, Chicago.

6. Porcelain Technology. Paper by H. J. Goslee. Discussion to be opened by Dr. J. Q. Byram, Indianapolis; Dr. Ambler Tees, Philadelphia; Dr. L. E. Custar, Dayton; Dr. H. L. Banshaf, Milwaukee; Dr. J. F. Ross, Toronto.

7. The Dental Curriculum. Paper by Dr. Geo. E. Hunt, Indianapolis. Discussion to be opened by Dr. G. V. Black; Dr. J. B. Willmott.

8. How Shall Quizzes be Conducted? Symposium by Dr. F. D. Weisse; Dr. R. H. Nones; Dr. L. P. Bethel.

9. Exhibition of Recent Teaching Appliances. Dr. W. G. Foster, Baltimore; Dr. L. S. Tenny, Chicago.

W. H. WHITSLAR, *Chairman Executive Committee.*

